# The Aging Urban Brain: Analyzing Outdoor Physical Activity Using the Emotiv Affectiv Suite in Older People

**DOI:** 10.1007/s11524-017-0191-9

**Published:** 2017-09-11

**Authors:** Chris Neale, Peter Aspinall, Jenny Roe, Sara Tilley, Panagiotis Mavros, Steve Cinderby, Richard Coyne, Neil Thin, Gary Bennett, Catharine Ward Thompson

**Affiliations:** 1 Stockholm Environment Institute, Environment Department, University of York, York, England; 2 School of Built Environment, Heriot-Watt University, Edinburgh, Scotland; 3 Center for Design and Health, School of Architecture, University of Virginia, Charlottesville, VA USA; 4 OPENspace Research Centre, Edinburgh College of Art, University of Edinburgh, Edinburgh, Scotland; 5 ETH-Centre, Singapore, Singapore; 6 Edinburgh School of Architecture and Landscape Architecture, University of Edinburgh, Edinburgh, Scotland; 7 School of Social and Political Science, University of Edinburgh, Edinburgh, Scotland; 8 The Stats People, Sevenoaks, Kent, England UK

**Keywords:** EEG, Mobility, Urban, Emotiv, Older adults, Green space

## Abstract

This research directly assesses older people’s neural activation in response to a changing urban environment while walking, as measured by electroencephalography (EEG). The study builds on previous research that shows changes in cortical activity while moving through different urban settings. The current study extends this methodology to explore previously unstudied outcomes in older people aged 65 years or more (*n* = 95). Participants were recruited to walk one of six scenarios pairing urban busy (a commercial street with traffic), urban quiet (a residential street) and urban green (a public park) spaces in a counterbalanced design, wearing a mobile Emotiv EEG headset to record real-time neural responses to place. Each walk lasted around 15 min and was undertaken at the pace of the participant. We report on the outputs for these responses derived from the Emotiv Affectiv Suite software, which creates emotional parameters (‘excitement’, ‘frustration’, ‘engagement’ and ‘meditation’) with a real-time value assigned to them. The six walking scenarios were compared using a form of high dimensional correlated component regression (CCR) on difference data, capturing the change between one setting and another. The results showed that levels of ‘engagement’ were higher in the urban green space compared to those of the urban busy and urban quiet spaces, whereas levels of ‘excitement’ were higher in the urban busy environment compared with those of the urban green space and quiet urban space. In both cases, this effect is shown regardless of the order of exposure to these different environments. These results suggest that there are neural signatures associated with the experience of different urban spaces which may reflect the older age of the sample as well as the condition of the spaces themselves. The urban green space appears to have a restorative effect on this group of older adults.

## Introduction

There is a large body of evidence, as reviewed by Velarde et al. [1, 2]; suggesting people generally have a preference for viewing natural over urban environments. Additionally, walking in nature has been shown to be beneficial for both well-being [3–5] and cognition [6, 7]. The literature suggests that the difference between directed (top-down) attention, where an environment demands increased cognitive effort, such as a busy road crossing, and involuntary (bottom-up) attention, where features of an environment are interesting as opposed to demanding, may contribute the mechanism for the beneficial effect of nature [8]. This aligns with Attention Restoration Theory [9–11] which posits that natural spaces have a restorative effect against cognitive fatigue. What is currently less well understood is the relationship between the environment and brain activity in older people. The neuroimaging literature suggests that there are specific neural signals that are observable when viewing images of a given environment as well as during real-time immersion physically in an environment. However, to date, the majority of this research has concentrated on younger participants whereas this study focusses on older people.

To date, in laboratory settings described in the academic literature, the comparisons between urban and green/natural spaces have typically been made using a clear contrast between built urban spaces and scenic rural spaces [1, 2], or at least have not differentiated between quieter urban spaces (such as residential spaces) and busy built urban spaces. There is therefore a need to understand the effects of different built urban spaces in real-world settings that may be qualitatively different in more subtle ways than a simple urban/rural contrast.

Functional magnetic resonance imaging (fMRI) has shown distinct networks of activation associated with viewing urban and rural scenes [8, 12] and demonstrated that these networks could be mediated by emotional preference [13]. In addition, reductions in resting state activation were found in the subgenual prefrontal cortex after a 90-min walk in nature as compared with a 90-min walk in an urban environment [3]. Electroencephalography (EEG) has shown that observing static rural images can induce increases in alpha (8–13 Hz) neural activity [14, 15] associated with relaxation [16].

Laboratory data using a mobile electroencephalography (EEG) headset (Emotiv EPOC+; validated for both laboratory and outdoor settings [17, 18]) showed outputs that corresponded with self-reported measures on attractiveness, willingness to visit, valence and arousal of static images of landscapes or urban scenes [19]. This study used data from the Affectiv Suite, Emotiv proprietary software, which analyzes EEG from distinct brain activity patterns and allocates a label defining an emotional parameter (‘frustration’, ‘excitement’, ‘engagement’, ‘meditation’, ‘long term excitement’). These parameters have been shown to accurately track different stages of learning in various scenarios [20]. Based on the results from a previous study [21] and discussions with the manufacturer, ‘engagement’ has been defined as immersion or interest, ‘excitement’ as high arousal, ‘frustration’ as negative valence and ‘meditation’ as a low arousal, rested state. Landscape scenes were predicted by increased levels of ‘meditation’ and lower levels of self-reported subjective arousal. The Affectiv Suite was further used in this pilot study to interpret implied brain activity associated with walking sequentially in a city through a quiet urban shopping street, then a green space and then a busy commercial district (urban busy) in a young (mean age 30.08 years) participant group (*n* = 12) [21]. The results showed lower levels of ‘frustration’, ‘engagement’ and ‘excitement’, and higher ‘meditation’ when moving into an urban green space and higher levels of ‘engagement’ when moving out of it into a busy-built urban street. However, the walk settings were experienced sequentially so it is unclear if the outcomes are products of the differential settings, walking over time, or the novelty of undertaking a mobile EEG study in a public place. The purpose of this study then is not to replicate the findings of the previous pilot, rather improve on the experimental design and test this improved protocol in an older participant group.

### Aims

Our current study aimed to understand the impact of the urban environment on neural activity using mobile EEG with older participants. Based on earlier findings using the Emotiv parameters [21], it was hypothesized that:

1. Walking in a busy urban street would be associated with comparatively higher levels of ‘frustration’, ‘excitement’ and ‘engagement’.
2. Walking in an urban green space would be associated with comparatively higher levels of ‘meditation’ and lower levels of ‘engagement’.
3. Walking in a quiet urban residential environment would be associated with comparatively higher levels of ‘engagement’, ‘excitement’ and ‘frustration’, but this effect would not be as pronounced as that found in the busy urban street.

## Method

### Participants

The participants were healthy adults aged 65 years and over (*N* = 95, mean age = 76.55 years, standard deviation = 8.15, range = 65–92 years) and were recruited by purposive sampling methods to ensure they met the inclusion criteria. All participants scored over 27 on the Mini-Mental State Exam (MMSE), suggesting that the participants were cognitively alert, scoring above Folsteins threshold of 24 for cognitive impairment [22]. Furthermore, the participants all reported being able to walk, unassisted, for at least 20 min, taken as a measure of physical ability to walk the assigned route. Exclusion criteria for study participation included visual impairments, chronic mental illness and a history of epileptic or psychiatric disorders. Ethical approval for the study was provided by the University of Edinburgh, Edinburgh College of Art Ethics Committee. To account for brain hemispheric differences [23], all participants in this study were right handed.

### Experimental Design and Procedures

All participants were screened via a phone conversation with the research team to ensure they fulfilled the inclusion criteria before being invited to undertake a practice session. This session served as an opportunity to demonstrate the EEG headset (described below) while familiarising participants with the route they would take (also described in the next section) during the experimental session. This was achieved by showing participants a 15-min video of the route. This was important as it meant participants subsequently did not need to refer to maps or consult the researchers for wayfinding and also generated familiarity with wearing the headset.

The experimental session was undertaken on a separate day when participants were equipped with a backpack to store the acquisition computer as well as having the EEG headset calibrated prior to commencing the walk. The participants were then instructed to walk on their assigned route at their own pace—meaning, walking times vary between participants (10–15 min on average)—understanding that a member of the research team was following behind for safety purposes. All experimental sessions were conducted in the morning to ensure time of day effects was kept to a minimum.

### Routes

**Fig. 1:**
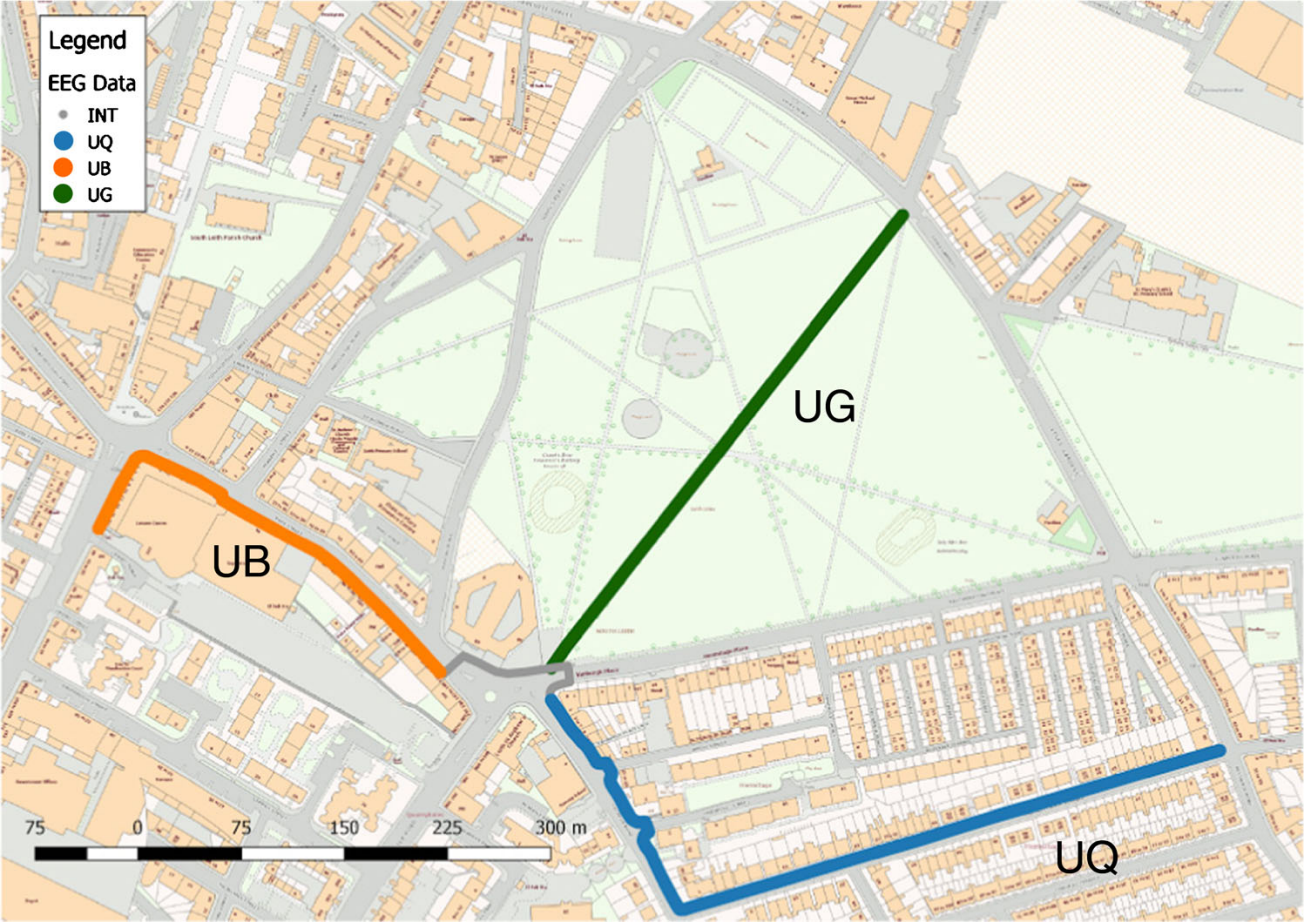
Map of the walking routes undertaken by participants (walking in one of six possible scenarios)

The participants walked through one of six walk scenarios, as indicated on the map in Fig. 1. The study site was based in Leith, an historic but deprived urban neighbourhood in Edinburgh, Scotland, and selected due to the proximity of green, quiet and busy urban spaces as well as a reasonably flat gradient to ensure participants could undertake the route without excessive exertion. Figure 1 indicates an interchange zone, in grey, between the green space and the busy- and quiet-built spaces, where participants had to cross a busy road junction. The busy road crossing was not modelled in the analysis due to the unpredictable nature of the conditions around the pedestrian crossing.

**Fig. 2:**
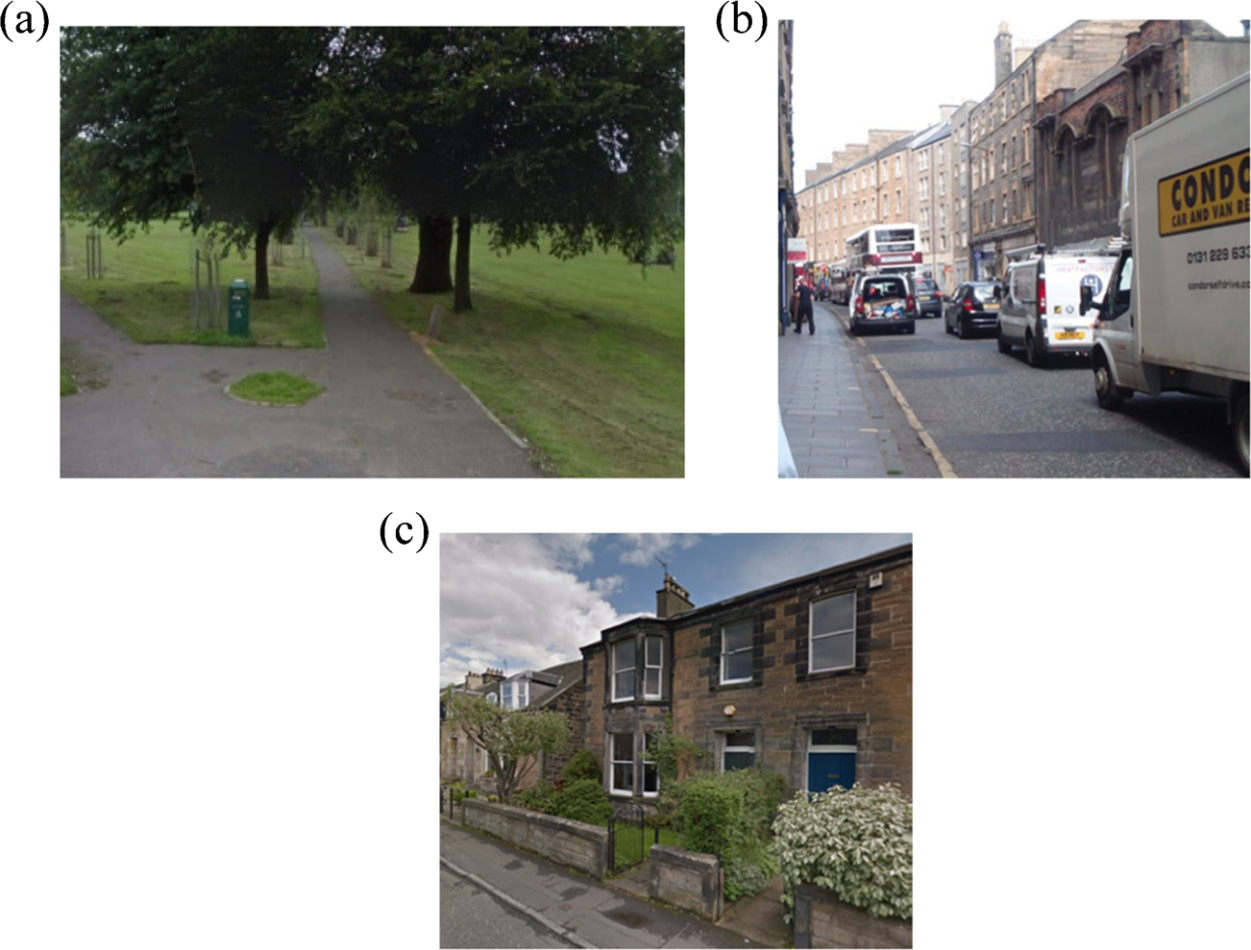
Street views of the three walking environments. **a** Urban green. **b** Urban busy. **c** Urban quiet (Photo credit: OPENspace Research Centre)

Figure 2a, b and c shows images of each of the three environments for walking. The participants were required to walk sequentially between two of these environments, with six possible routes generated from this design:

1. Urban busy to urban green (*n* = 20)
2. Urban busy to urban quiet (*n* = 14)
3. Urban green to urban busy (*n* = 20)
4. Urban green to urban quiet (*n* = 13)
5. Urban quiet to urban busy (*n* = 14)
6. Urban quiet to urban green (*n* = 14)

We describe green space as an area with a predominance of vegetated and non-built surfaces (including grass and trees). We describe urban busy spaces as having a predominance of buildings, paved areas and a commercial street frontage which attracts a high footfall and considerable vehicular traffic. Finally, we describe urban quiet spaces as having a predominance of buildings, some front gardens and paved areas, but these are largely residential and, as a result, do not attract a high footfall or prevalence of vehicular traffic.

### EEG Data Acquisition

Brain electrical activity was recorded non-invasively from the scalp using the Emotiv EPOC+ EEG headset with 14 channels corresponding to the international 10–20 position system (AF3, AF4, F3, F4, F7, F8, FC5, FC6, T7, T8, P7, P8, O1 and O2). P3 and P4 acted as reference electrodes. Electrode impedances were kept below 5 kΩ and signals were internally sampled at 1024 Hz before being internally down sampled to 128 Hz per channel and sent via Bluetooth to the acquisition computer. The Affectiv Suite creates a different profile for each individual to account for potential differences at the neural level, and then interprets the EEG activity from the available channels into the four emotional parameters: ‘excitement’ (short-term arousal), ‘frustration’, ‘engagement’ and ‘meditation’. These parameters were normalized for each individual and scaled as values between 0 and 1, which allow between-subject comparisons, at each sampling point. This process results in approximately seven samples per second (7 Hz). Data inspection indicated that in the majority of cases, there was flat-lining in the ‘meditation’ channel; therefore, the presented analysis only assesses the effect of the environment on levels of ‘engagement’, ‘excitement’ and ‘frustration’. We defined flat-lining as data which did not deviate from a particular value (or limited range of values) for the entire duration of a participant’s walk. Table 1 presents working definitions of each of the Affectiv Suite parameters as understood by the authors from previous research or discussions with representatives of Emotiv.

**Table 1:** Descriptions of the Affectiv Suite outputs

Affectiv Suite parameter	Working description
Engagement	Likened to immersion, interest or directed attention
Excitement	Correlated with classic arousal indicators such as increased heart rate and blood flow
Frustration	Associated with stress and disappointment/negative valence
Meditation	No emotional context, purely correlated with low arousal and a rested state

### Data Analysis

In order to analyze the data, one mean for each individual per walking segment was generated for each of the paired settings in a walk scenario for each of the three emotional parameters available via the Emotiv Affectiv Suite software. These means were standardized by subtracting the group mean from the raw mean for each individual and dividing this by the standard deviation of the group mean. This data set was subsequently analyzed using a form of logistic high-dimensional correlated component regression (CCR) able to deal with smaller samples where *p* (number of predictors) is greater than *n* (number of cases) as well as repeated measures multicollinearity [24, 25]. We assessed difference scores between environmental conditions for each of the six walking routes generated by a calculation of *x* = (walk A − walk B) at the participant level for each of the Affectiv Suite emotional parameters. Outliers were identified and amended using a criterion of *z* = 2.5 (i.e. high difference outliers which might be unduly influential were brought back to the highest value within 2.5 standard deviations [26]). These final differences scores were used in the regression analysis.

## Results

### Comparison of Walking between Urban Busy and Urban Green Environments

The high dimensional CCR analysis shown in Table 2 indicates that there were statistically significant differences between urban busy (UB) and urban green (UG) environments with regards to levels of ‘engagement’ and ‘excitement’. There was no effect of the changing environment on levels of ‘frustration’ in either route.

**Table 2:** Logistic CCR outputs for the UB and UG routes

Model fit	Training	Cross-validation	Standard error
*R* ^2^	.096	.014	.015
Area under curve (AUC)	.663	.531	.052
Accuracy	.630	.540	.050
Predictors	Standardised coefficient	Out of sample frequency (*n* = 100 runs)	Pratt coefficient of relativeimportance to the model
Engagement	1.2793	95	14%
Excitement	−.9859	80	86%

*AUC* area under the curve

**Fig. 3:**
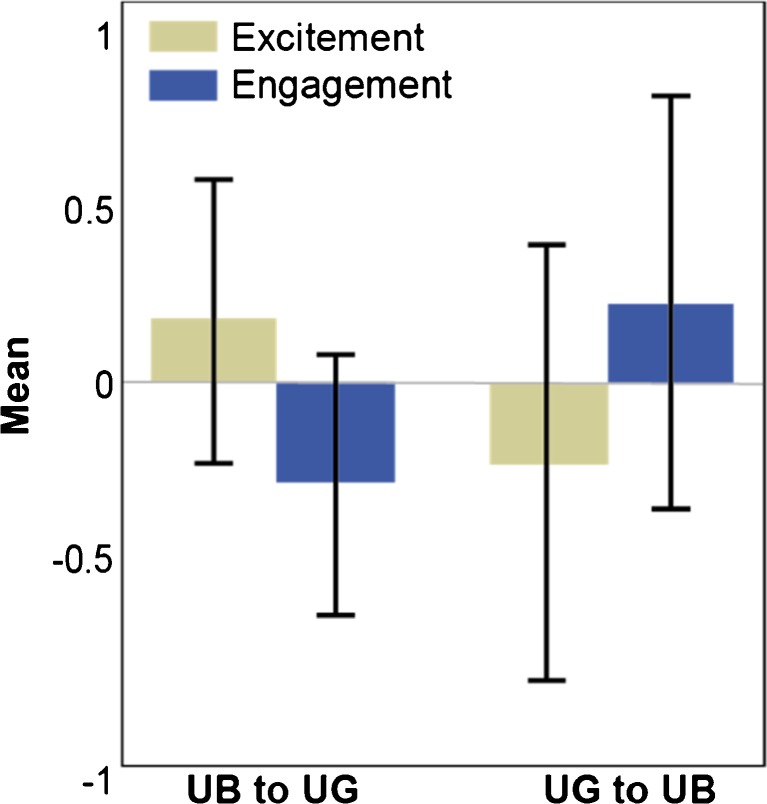
‘Excitement’ and ‘engagement’ difference scores for each walking condition showing that ‘excitement’ is greater in the UB setting than the UG setting. ‘Engagement’ follows a reverse pattern

The ‘model fit’ section of Table 2 shows the value of *R* [2] from cross-validation along with its standard error (SE) showing a small to medium effect size. The standardised coefficients from the usual regression output are shown, indicating the rank order of predictors in the model. The positive and negative signs of the standardised coefficients in Table 2 associated with ‘engagement’ and ‘excitement’ levels are given context in Fig. 3. The figure shows the differences in ‘excitement’ and ‘engagement’ in going from the first to the second part of each walking scenario. A positive value indicates levels for that parameter are greater in the first part of the walk and a negative value indicates levels for that parameter are greater in the second part of the walk. This means that ‘excitement’ is higher in UB than in UG and that ‘engagement’ is lower in UB than in UG. However, for the walk in the reverse direction (UG to UB), the results are reversed. ‘Excitement’ is negative, meaning that it is lower in UG than UB; ‘engagement’ is positive, meaning it is higher in the UG than the UB section. The Pratt coefficient shows that ‘excitement’ contributed 86% to the model.

### Comparison of Walking between Urban Busy and Urban Quiet Environments

The high dimensional CCR analysis shown in Table 3 indicates that there were statistically significant differences between UB and UQ environments with regard to levels of ‘excitement’. There was no effect of the changing environment on levels of ‘engagement’ or ‘frustration’ in either route.

**Table 3:** Logistic CCR outputs for the UB and UQ routes

Model fit	Training	Cross-validation	Standard error
*R* ^2^	.220	.050	.046
AUC	.786	.473	.081
Accuracy	.647	.569	.065
Predictors	Standardised coefficient	Out of sample frequency (*n* = 100 runs)	
Excitement	−1.975	91	

*AUC* area under the curve

**Fig. 4:**
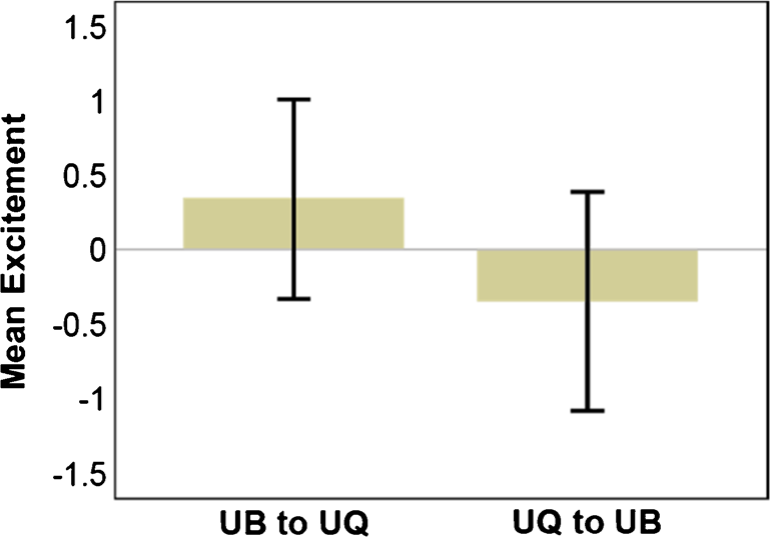
‘Excitement’ difference scores for each walking condition showing that ‘excitement’ is greater in the UB setting than the UQ setting

The ‘model fit’ section of Table 3 shows the value of *R* [2] from cross-validation along with its standard error (SE), showing a small to medium effect size. The standardised coefficients from the usual regression output are shown, indicating the only predictor in the model is ‘excitement’. The negative sign of the standardised coefficients in Table 3 associated with the ‘excitement’ parameter is a given context in Fig. 4. The figure shows the differences in ‘excitement’ going from the first to the second part of each walking scenario. This means that, going from UB to UQ, ‘excitement’ is higher in UB than in UQ. However, for the walk in the reverse direction (UQ to UB), the results are reversed. ‘Excitement’ is negative, meaning that it is lower in UQ—the first walk component—than UB.

### Comparison of Walking between Urban Green and Urban Quiet Environments

The high dimensional CCR analysis shown in Table 4 indicates that there were statistically significant differences between UG and UQ environments with regard to levels of ‘engagement’ and ‘frustration’. There was no effect of the changing environment on levels of ‘excitement’ in either route.

**Table 4:** Logistic CCR outputs for the UG and UQ routes

Model fit	Training	Cross-validation	Standard error
*R* ^2^	.296	.053	.061
AUC	.773	.668	.093
Accuracy	.789	.689	.095
Predictors	Standardised coefficient	Out of sample frequency (*n* = 100 runs)	Pratt coefficient of relative importance to the model
Engagement	3.737	97	66%
Frustration	2.932	95	34%

*AUC* area under the curve

**Fig. 5:**
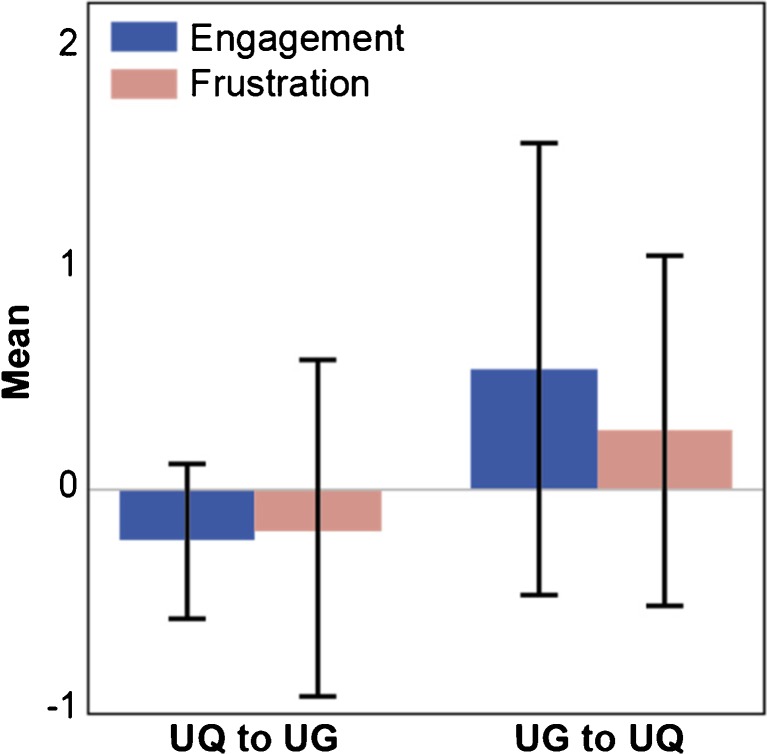
‘Engagement’ and ‘frustration’ difference scores for each walking condition showing both parameters are greater in the UG setting than the UQ setting

The ‘fit’ section of Table 4 shows the value of *R* [2] from cross-validation along with its standard error (SE), showing a small to medium effect size. The standardised coefficients from the usual regression output are shown, indicating the rank order of predictors in the model. The positive signs of the standardised coefficients in Table 4 associated with ‘engagement’ and ‘frustration’ levels are given context in Fig. 5. The figure shows the differences in ‘engagement’ and ‘frustration’ in going from the first to the second part of each walking scenario. For the first scenario (UQ to UG), the change is negative for both ‘engagement’ and ‘frustration’. This means that both ‘engagement’ and ‘frustration’ levels are lower in UQ than those in UG. However, for the walk in the reverse direction (UG to UQ), the situation is reversed, with both ‘engagement’ and ‘frustration’ being positive, meaning they are both higher in UG—the first walk component—than UQ. The Pratt coefficient shows that ‘engagement’ contributed 66% to the model, while ‘frustration’ contributed 34% to the model.

## Discussion

The results show higher levels of ‘excitement’ in urban busy settings compared with both green and urban quiet walks, higher levels of ‘engagement’ in the green setting compared with urban busy and urban quiet and higher levels of ‘frustration’ in the green setting when compared with the urban quiet setting. These results add to the growing literature regarding neural change associated with experiencing changing environments.

Our first hypothesis regarding the urban busy environment posited comparatively higher levels in ‘excitement’, ‘engagement’ and ‘frustration’ from walking in the urban busy walking scenarios. We found higher levels of ‘excitement’ in the urban busy location, as hypothesised, against both the green and urban quiet settings. However, contrary to our hypothesis, higher levels of ‘engagement’ or ‘frustration’ were not associated with walking in an urban busy location. ‘Excitement’ may be indicative of increased levels of top-down attention previously associated with viewing urban images [8]. This aligns with the spatial context in that the urban busy route has an increased amount of both vehicular and pedestrian traffic and participants also encountered increased obstacles in the path (e.g. refuse bins, other pedestrians) compared with the other settings. Given that all our participants were 65 years or over (mean age 77), there may be age-associated difficulties in spatial navigation [27, 28], which require increased levels of top-down attention to negotiate this section of the walk. The study neighbourhood used here was less affluent than that used in our previous research, reflected in poorer quality paths and paving, and as such may require greater levels of cognitive alertness in order to navigate. Poor path quality may contribute to increased motion artefacts from the participants. However, poor path quality is deemed to be consistent between route types (i.e. urban busy, urban quiet and green spaces). Further work should then focus on the impact of path quality on mobile EEG, in particular differences between different path qualities and if there are differences in varied public spaces. The finding that ‘excitement’ is not present in the model contrasting the green and urban quiet scenarios suggests that the urban busy setting induces neural behavior that is unique to this particular setting. The relationship between the ‘excitement’ and ‘engagement’ parameters, shown clearly between the urban busy and urban green segments, may be reflective of the different neural processing required for top-down and bottom-up attention.

Our second hypothesis regarding the urban green environment stated we would see comparatively higher levels of ‘meditation’ and lower levels of ‘engagement’ from walking in the green walking scenarios. We were unable to investigate the role of the ‘meditation’ channel for the reasons outlined earlier, but this study shows increases in ‘engagement’—as opposed to decreases shown previously [21]—in the urban green condition over both the urban busy and urban quiet conditions. In our earlier paper [21], ‘engagement’ was interpreted as directed attention due to increases in cognitive attention associated with transitioning from green to urban busy. However, it is important in this context to understand *how* this attention is being directed. There are two mechanisms for attentional processing: bottom-up and top-down. Neuroimaging research has suggested that there are distinct networks of neural activity associated with top-down and bottom-up processing [29] and that, in terms of mental well-being, there is a benefit of bottom-up processing as it is associated with involuntary attention [30]. Green environments are said to influence our involuntary attention as compared to urban environments that demand our attention directly. This effect is described in environmental psychology as ‘soft fascination’ [10], a process that is associated with restorative health effects, including relief from fatigue, stress and low mood [9]. Conversely, top-down processing is associated with increased directed attention and subsequent fatigue [31]. Recent fMRI research has indicated that viewing urban images is associated with an active network similar to that present in top-down attentional processing, while viewing images of green scenery is associated with an active network reflective of bottom-up processing [8]. Perhaps, the results presented, showing higher level of ‘engagement’ in the green condition in this study, are indicative of increased bottom-up processing. Previous EEG research has shown increases in alpha activity, associated with increased relaxation, when viewing of static green images [14, 15], so we might hypothesise that alpha changes may be involved with ‘engagement’, given the correlation between increases in alpha associated with green space in a laboratory setting and increases in ‘engagement’ walking in green spaces presented here. Further analysis is required, perhaps primarily at the laboratory level, to understand the relationship between the Affectiv Suite outputs, the traditional raw EEG outputs and the psychological and physiological implications of these.

We also see comparatively higher levels of ‘engagement’ in the green condition when transitioning to or from an urban quiet condition. This again suggests that the green environment used in this study has more restorative properties than those in the built urban space, despite this space being comparatively quiet in this instance. There was no difference in the level of ‘engagement’ in the urban quiet vs. urban busy condition, as the effect of ‘engagement’ is limited to the green space. The urban quiet setting used in this study was made up of largely residential properties with small front gardens; this mixture of ‘built’ and ‘green’ spaces may lead to increases in soft fascination, as discussed earlier [9, 10, 30], but these effects are not significant in comparison with exposure to urban busy spaces. Previous research has suggested that quiet residential spaces can be beneficial for the well-being in residents, based on the attractive soundscapes and associated tranquillity present in these spaces [32, 33], suggesting that the urban quiet setting encourages soft fascination, but this beneficial effect has not been shown to be significant here.

An alternative explanation of our findings could be that ‘engagement’ in this context relates to increased levels of directed attention associated with negotiating poorer quality paving in the green space when compared with the paving in the urban busy and urban quiet settings. Research has suggested that poor paving is a barrier for older people in engaging with outdoor environments [34]. However, in this case, while the paving quality may be poor in the green space compared with that of the urban busy and urban quiet spaces, the urban spaces have an increased need for increased vigilance due to road crossings, increased pedestrian and vehicular traffic and increased levels of noise, combining to create potential ‘sensory overload’. Generally, maintenance of outdoor spaces is seen to be vital in order for older people to benefit from them [35]. Therefore, more investigation may be required to understand the link between the ‘engagement’ parameter and urban spaces with regard to the barriers that each walking scenario may offer.

We also found higher levels of ‘frustration’ in the green condition compared with the urban quiet condition. This is the only model in which ‘frustration’ is shown as a significant predictor, not appearing in either of the models that include urban busy, contrary to the hypotheses. We have previously defined ‘frustration’ as being associated with negative valence [21], so it is unclear why this is present in this particular model. That it is not present in the urban green and urban busy model suggests that there is something particular about the difference between the urban green and urban quiet condition.

There is a large body of literature that suggests that green environments have a restorative effect [10, 11] and that this effect can be seen when walking in green space in both healthy [4, 6] and clinical [5, 7] populations. Recent research has suggested that even a short exposure to a static green scene is associated with improved behavioral performance on a sustained attention task when compared to short exposure to an image of a built urban environment devoid of nature [36]. The results presented here suggest that this restorative effect may extend to the quiet urban condition selected for this study. Our study could have investigated the effect of restoration further by analysis of the ‘meditation’ output, but as previously discussed, this channel was removed from the analysis due to flat-lining of this data.

Our third hypothesis posited that there would be higher levels of ‘excitement’, ‘engagement’ and ‘frustration’ in walking in the urban busy condition. There does not appear to be any urban quiet-specific difference that would suggest there is a neural pattern that is a product of only the urban quiet setting, unlike that shown with ‘excitement’ in the urban busy setting or ‘engagement’ in the urban green setting. This space is perhaps reflective of a mixture of the urban busy setting, in that the quality of the paths is similarly next to vehicular traffic, and the green setting where there is less vehicular and pedestrian traffic as well as an increase in greenery in some front gardens.

The previous, pilot mobile EEG study, also conducted in Edinburgh, albeit in a different and more affluent neighbourhood and in a younger, smaller sample, using Affectiv Suite data, suggested a different pattern of brain activity to that found here [21]. The previous data indicated that ‘frustration’ and ‘engagement’ decreased when transitioning from a quiet urban environment into a green environment, with ‘engagement’ increasing again moving from the green space to a busy urban space. These results are not replicated in the current study, which may be indicative of the particular urban environments in new routes undertaken, or in the responses of older aged participants. Other research suggests there are age-related changes in neural activation [37, 38] which could explain the different findings shown here.

The key finding from this study is the relationship between the two, Affectiv Suite-defined, emotional parameters of ‘engagement’ and ‘excitement’ in the comparison of the urban busy and urban green routes. ‘Excitement’ levels appear to be significantly higher in the urban busy environment when compared with those of the green environment, as hypothesised. The opposite is true of ‘engagement’ in that ‘engagement’ levels are higher in the green environment when compared with those of the urban busy environment. This is the opposite of what we hypothesised. It suggests there may be a relationship between these two parameters in terms of the neural processing that underpins the Affectiv Suite definition of these terms. There was no statistically significant change in levels of ‘frustration’ between the two walking conditions.

### Limitations

Due to the intellectual property rights of Emotiv, we do not have access to details on which particular EEG signature underlies each of the Affectiv Suite outputs. Therefore, while the results here suggest neural change associated with varying urban environments, it is important to recognise that only an assessment of the EEG data using given frequency bands can fully illuminate the neural basis of this change [39]. Future research may also focus on providing a quantitative comparison of effects on both young and old participants to demonstrate if there are age-associated changes in neural activity linked with experience of the urban environment. Furthermore, there may be interest in looking at gender differences in responses to urban environments, as these differences could influence the way we perceive and design fully inclusive public spaces. This was unfortunately beyond the scope of this paper, as further dividing the participant group into smaller sub-groups would likely have had a detrimental impact on the statistical analyzes.

While the method and protocol used in this study is useful for understanding differences between settings, future studies could modify this to look at differences within settings. For example, assessing the specific ‘ingredients’ within an urban space (e.g. number of people, street connectivity, architecural variation in facades) and the interaction with health and wellbeing outcomes, as measured by neural activity, is of interest. Recent research has presented a tool for describing public spaces using both space syntax and isovist analysis to understand the connectivity of street segments and shape and size of open spaces, both predicting levels of stress associated with each [40]. Indeed, in our study, the urban green setting appears to be highly connected with multiple paths and roads adjacent to the space, unlike the urban quiet setting, implying a potentially higher average of pedestrian thoroughfare [41]. Utilising additional analyzes, and collecting more real-time data (e.g. pedestrian counts during testing sessions), would give a richer understanding of the dynamics of the different settings. Our recent work suggested that a subset of participants from this study found the urban green setting relaxing and peaceful [42], but that could change at different times of the day.

Additionally, it would be beneficial to understand differences that may occur between laboratory and ‘mobile’ EEG recordings. By its very nature, mobile EEG is much more prone to known EEG motion artefacts from both head and eye movements [43]. However, it is unlikely that these artefacts are due to the makeup of the walk settings detailed here, rather they would be present throughout the recording sessions. Therefore, it is unlikely that these walk settings generate consistent or systematic blinking rates that could result in differences between the Affectiv Suite parameters shown in the abovementioned data.

## Conclusion

This research shows varying levels of neural activity in different urban environments while walking at a specific set of locations in central Edinburgh. The study has shown higher levels of ‘engagement’ associated with walking in an urban green space compared to both a busy urban commercial street and quiet residential area, as well as higher levels of ‘excitement’ walking in a busy urban space compared to both a green space and quiet urban space. These findings are consistent with restorative theory. To the authors’ knowledge, this is the first time such an effect has been shown using a sample of older, healthy adults. The protocol developed here is adaptable and could be further applied toward improving understanding of how best to utilise urban and green spaces for efficient age-friendly urban design. These results indicate the potential beneficial effects of walking in a green environment in an urban setting. Further investigation is required to understand the exact neurological processes that underpin these changes.
